# INEQUALITIES IN FRAILTY BY SOCIO-ECONOMIC POSITION: IT’S ALL IN THE TIMING

**DOI:** 10.1093/geroni/igz038.829

**Published:** 2019-11-08

**Authors:** Carol Jagger, Mark D Hayward

**Affiliations:** 1 Newcastle University, Newcastle upon Tyne, United Kingdom; 2 University of Texas at Austin, Austin, Texas, United States

## Abstract

A number of studies have found that higher socio-economic position (SEP) appears protective of becoming frail. However, not only can SEP be defined in early, mid or late life, by education, occupational status or income/material disadvantage respectively, but frailty may occur in the young old as well as the very old. Do the same measures of SEP reflect inequalities in frailty in the young old as the very old? Does it matter when in the lifecourse SEP is measured? Have inequalities in frailty between SEP groups changed across the generations of older people? We seek to answer such questions from cohorts across the spectrum of later life. The first presentation, from the 1958 British Birth Cohort Study, examines the association between early-life SEP and frailty at age 50 and whether this association is due to continued disadvantage into mid-life. The second presentation moves to very old age, examining the role of early, mid and late life disadvantage on the progression of frailty between ages 85 and 90 in the Newcastle 85+ cohort. The third presentation, based on the electronic health records of adults aged 75 years and over in England, focuses on whether SEP modifies frailty trajectories in the last year of life. The final presentation examines whether SEP inequalities in frailty have changed over different generations of older people, and utilises data from the Cognitive Function and Ageing Studies. Together these presentations increase understanding of which SEP groups should be targeted for interventions to reduce frailty throughout later life.

